# Clinical efficacy of cell-free fat extract and its effects on bone marrow edema in patients with early to mid-stage knee osteoarthritis: a clinical trial in comparison with hyaluronic acid

**DOI:** 10.1186/s13018-025-05543-3

**Published:** 2025-02-09

**Authors:** Changchun Zhang, Yuanshi Lu, Yuanxia Huang

**Affiliations:** The First Affiliated Hospital of Xinxiang Medical University, Weihui, Henan China

**Keywords:** Cell-free fat extract, Hyaluronic acid, Early to mid-stage knee osteoarthritis, Bone marrow edema

## Abstract

**Background:**

Previous studies have shown that hyaluronic acid can delay the progression of knee osteoarthritis. Existing research has extracted a bright red fluid called cell-free fat extract from human adipose tissue, which may play an important role in delaying the progression of osteoarthritis. By comparing with intra-articular injection of hyaluronic acid, this study aimed to evaluate the effects of intra-articular injection of CEFFE on both clinical efficacy and the reduction of bone marrow edema in patients with early to mid-stage knee osteoarthritis.

**Methods:**

A total of 48 patients with KOA (Kellgren-Lawrence grade II-III) symptoms were randomly divided into CEFFE group (24 cases) and HA group (24 cases). The patients in the CEFFE group received five injections of CEFFE (2 ml, 1 time/week), and the patients in the HA group received five injections of HA (2 ml, 1 ml/10 mg, 1 time/week). All the patients underwent clinical assessments using rating scales, including VAS, WOMAC and Lysholm Knee Score. These assessments were conducted at pre-treatment and at 3-week, 6-week, 3-month, and 6-month follow-up timepoints post-treatment. The clinical efficacy was evaluated at the 6-month follow-up after the treatment. The changes in subchondral bone marrow edema before and 6 months after treatment were assessed by grading BME on MRI of the affected knees.

**Results:**

A total of 52 knees from 46 patients were included in the final analysis. Comparison of VAS score, WOMAC score, and Lysholm score between the two groups revealed that the differences between pre-treatment and 3 weeks post-treatment were not statistically significant (*P* > 0.05). For the VAS score and WOMAC score at 6 weeks, 3 months, and 6 months post-treatment, the CEFFE group was lower than the HA group (*P* < 0.05). For the Lysholm score, the CEFFE group was higher than the HA group (*P* < 0.05). Compared with pre-treatment, VAS scores and WOMAC scores were lower and Lysholm scores were higher at all post-treatment time points (*P* < 0.05). At 6 months post-treatment, the clinical efficacy of the CEFFE group was significantly better than that of the HA group (*P* < 0.05). At 6 months post-treatment, MRI grading showed that subchondral BME was reduced to different degrees in both groups, with the reduction being more pronounced in the CEFFE group (*P* < 0.05).

**Conclusion:**

This study demonstrated that intra-articular injection of CEFFE into the knee joint could enhance the durability of tissue-specific cells (especially chondrocytes) and improve cellular metabolic processes, preventing the continued progression of osteoarthritis. Both CEFFE and HA were found to improve clinical symptoms and reduced subchondral bone marrow edema in the treatment of early to mid-stage knee osteoarthritis. However, CEFFE was more effective than HA in achieving these outcomes.

## Background

The knee joint is the predilection site of osteoarthritis [1]. Knee osteoarthritis (KOA) is a chronic disease characterized by progressive damage to the cartilage, subchondral bone, synovial tissues, and other intra-articular structures of the knee joint [2, 3]. The increasing incidence of knee osteoarthritis reduces people’s quality of life, increases disability and imposes a huge economic burden on society directly or indirectly [4, 5]. The clinical symptoms of knee osteoarthritis are mainly pain, swelling, stiffness and limited mobility of knee joint [6]. Among these, the pain is associated with an imbalance between anti-inflammatory cytokines and inflammatory cytokines within the intra-articular microenvironment of the knee joint [7, 8].

The treatment of knee osteoarthritis is categorized into surgical and conservative treatments. Surgical treatment is an important means for managing the end stage of knee osteoarthritis. However, patients with early to mid-stage knee osteoarthritis are more likely to choose conservative treatments, including physical exercise, weight loss, oral non-steroidal anti-inflammatory drugs (NSAIDs) or glucosamine. These treatments can provide temporary pain relief, but cannot repair cartilage damage or slow the progression of osteoarthritis [9–11]. Intra-articular injection of hyaluronic acid (HA) is widely used in the treatment of knee osteoarthritis [11]. Hyaluronic acid, a complex glycosaminoglycan secreted by cartilage within synovial fluid, enhances the elastic viscosity of intra-articular synovial fluid. It acts as a lubricant and shock absorber, protects intra-articular cartilage and promotes cartilage repair [12]. Therefore, Hyaluronic acid can temporarily relieve the symptoms of intra-articular pain, albeit with inconsistent clinical effects [13]. With the technological advances in tissue engineering and regenerative clinical medicine, several biological agents have been identified that exhibit anti-inflammatory, nutritive, angiogenic and immunomodulatory effects. The agents include platelet-rich plasma (PRP), stromal vascular fraction (SVF), autologous bone marrow cells (BMAC) and umbilical cord tissue (UC), all of which can serve as effective treatment options for early to mid-stage knee osteoarthritis [14–18]. After further exploration, their derivative reagents have been obtained, including platelet lysate (PL), allogenic platelet-rich plasma, growth factor concentrate (GFC), adipose-derived stromal cells (ADSC) and Wharton’s jelly (WJ), all of which are also effective in delaying the progression of KOA [7, 13, 17, 19, 20].

Recently, biological agents derived from adipose tissue have been presented as a promising alternative for the treatment of knee osteoarthritis. It has been found that micro-fragmented adipose tissue (MFAT), obtained from adipose tissue by mild mechanical treatment with the Lipogems system, is rich in pericytes and mesenchymal stem cells (MSCs). These cells release angiogenic, anti-inflammatory and immunomodulatory growth factors and cytokines [21, 22], which possess the ability to promote tissue regeneration and have great prospects in the treatment of knee osteoarthritis and other clinical applications [23–25]. A recent study has extracted a light red autologous fat extract from suctioned human adipose tissue after centrifugation and emulsification, referred to as Cell-Free Fat Extract (CEFFE). This extract has been found to contain a significant number of various cytokines that promote tissue regeneration and repair, as demonstrated by ELISA [26]. CEFFE can induce the migration, proliferation, differentiation, and stabilization of neovascular endothelial cells, promote angiogenesis, and reduce tissue ischemic injury. Additionally, it can promote the migration, proliferation and activation of fibroblasts [27], and enhance the antioxidant capacity of fibroblasts [28]. Therefore, based on the above biological properties of CEFFE, it can be clinically applied to treat cardiovascular diseases, enhance the survival rate of fat grafts, accelerate wound healing, preserve ovarian function, and improve fertility [26–31]. In contrast to its homologous MFAT, CEFFE is an acellular fluid that can exert therapeutic effects comparable to those of stem cells, avoiding the safety issues associated with cellular treatments. Studies have indicated that CEFFE can promote cartilage regeneration, decrease levels of inflammatory mediators, and eliminate excessive oxidative stress, thereby playing an important role in inhibiting or delaying the progression of early to mid-stage osteoarthritis [32]. However, there have been no studies on the intra-articular application of CEFFE in human joints and its clinical efficacy in treating osteoarthritis. Bone marrow edema (BME) is a common finding on MRI in patients with knee osteoarthritis, and some studies have shown that the size of the lesion area can predict the progression of osteoarthritis [33, 34]. Therefore, based on these findings, the present study was conducted to investigate the effects of intra-articular injection of CEFFE on both clinical efficacy and the reduction of bone marrow edema in patients with early to mid-stage knee osteoarthritis by comparing it with intra-articular injection of hyaluronic acid.

## Methods and materials

### Participant selection

In this prospective, randomized controlled trail, patients with early to mid-stage knee osteoarthritis who visited the orthopedic outpatient clinic of the First Affiliated Hospital of Xinxiang Medical University from September 2022 to January 2024 were selected. The study adhered strictly to the guidelines of the Declaration of Helsinki, and ethical approval was obtained from the Ethics Review Committee of the First Affiliated Hospital of Xinxiang Medical University (ID: EC-023–226). Written informed consents were obtained from participants. Participants were examined by experienced orthopedists at our hospital. Inclusion criteria: age range: 35–75 years old; had a medical history of pain, swelling and dysfunction in unilateral or bilateral knee joints; Kellgren-Lawrence grade II-III, or MRI manifestation of cartilage bone marrow edema, joint effusion, bone cysts, and cartilage damage; met the diagnostic criteria of primary knee osteoarthritis; voluntary participation in the trial with a signed informed consent form. Exclusion criteria: had a medical history of intra-articular surgical treatment or medication injection within the past 6 months; severe limb deformity (inversion > 5° or eversion > 5°); use of non-steroidal anti-inflammatory drugs within the last week; presence of comorbidities such as rheumatoid arthritis, hematologic disorders, coagulation disorders, diabetes mellitus, cerebro-cardiovascular diseases, infections or immunosuppression; presence of infections or active wounds on the skin of the knee joint; hemoglobin < 12 mg/dl or platelets < 150,000/mm^3^; presence of mental disorders, inability to cooperate with the trial or withdrawal from the test during its course.

### Randomization and enrollment

**Fig. 1:**
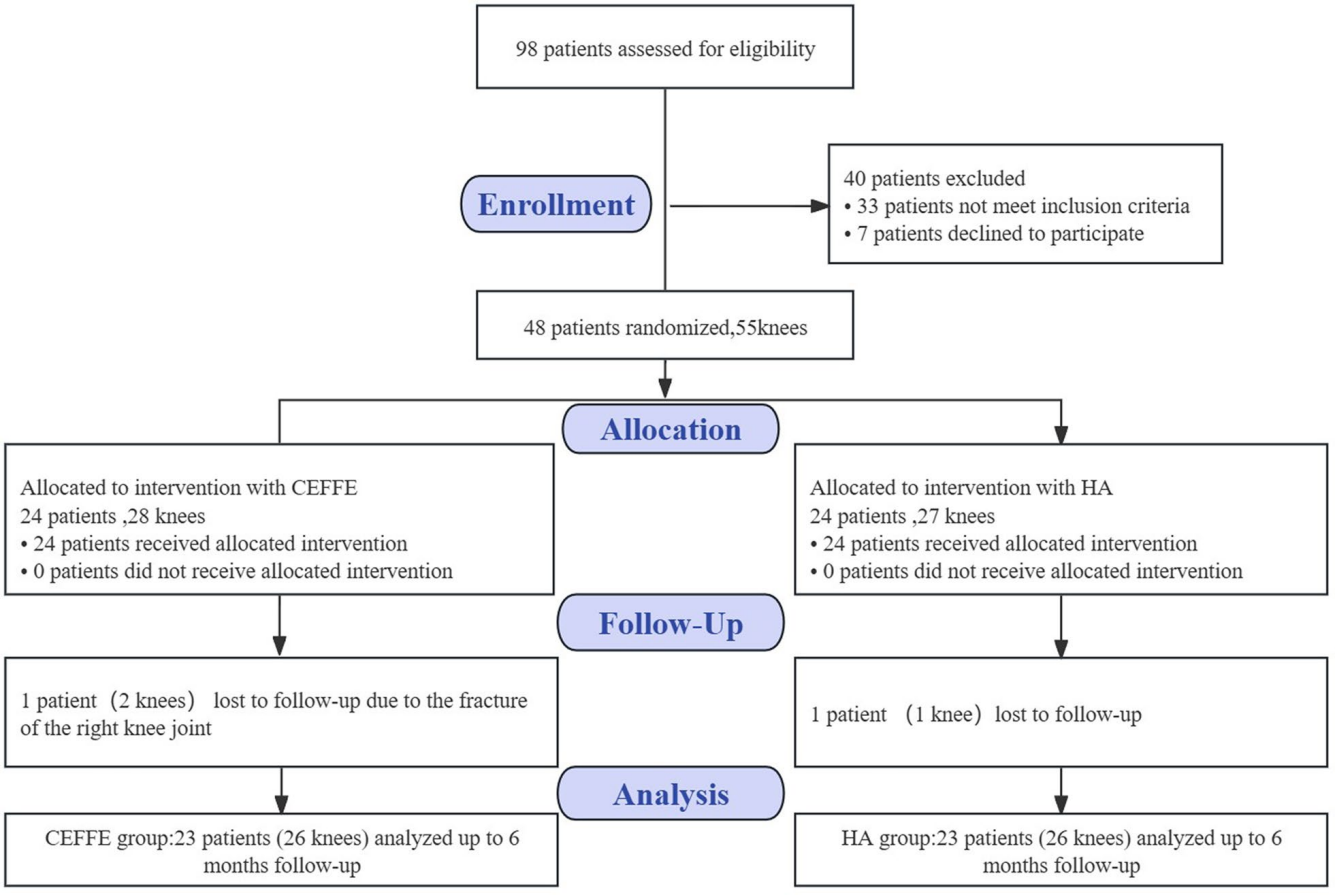
CONSORT (Consolidated Standards of Reporting Trials) flow diagram for the present study. CEFFE, cell-free fat extract; HA, hyaluronic acid

A total of 98 patients were evaluated before the surgery, including medical history, physical examination, laboratory tests (blood counts, ALT, AST, Cr, BUN, GFR, PT, PTT, HBsAg, HIV Ag/Ab, etc.) and imaging tests (weight-bearing anteroposterior and lateral radiographs of the knee joint, MRI of the knee joint). Based on the established inclusion and exclusion criteria, a total of 48 subjects with early to mid-stage knee osteoarthritis were enrolled. Using random allocation software, these subjects were randomized in a 1:1 ratio into an experimental group (CEFFE group) and a control group (HA group). Notably, four patients with bilateral knee osteoarthritis were involved in the CEFFE group and 3 in the HA group. During the 6-month follow-up period, one patient with bilateral knee osteoarthritis in the CEFFE group withdrew from the trial due to a fracture in the right knee; one patient with a unilateral knee osteoarthritis in the HA group was lost to follow-up. Consequently, a total of 46 patients were enrolled in the trial (n = 23), and 3 patients with bilateral knee osteoarthritis were involved in each group. Ultimately, 26 knees in each of the two groups were included in the trial (Fig. 1). The randomization process was conducted by a resident using allocation concealment, and a different resident, who was blinded to the subject assignments, was responsible for the pre-treatment and post-treatment follow-up of all subjects.

### Preparation of CEFFE

**Fig. 2:**
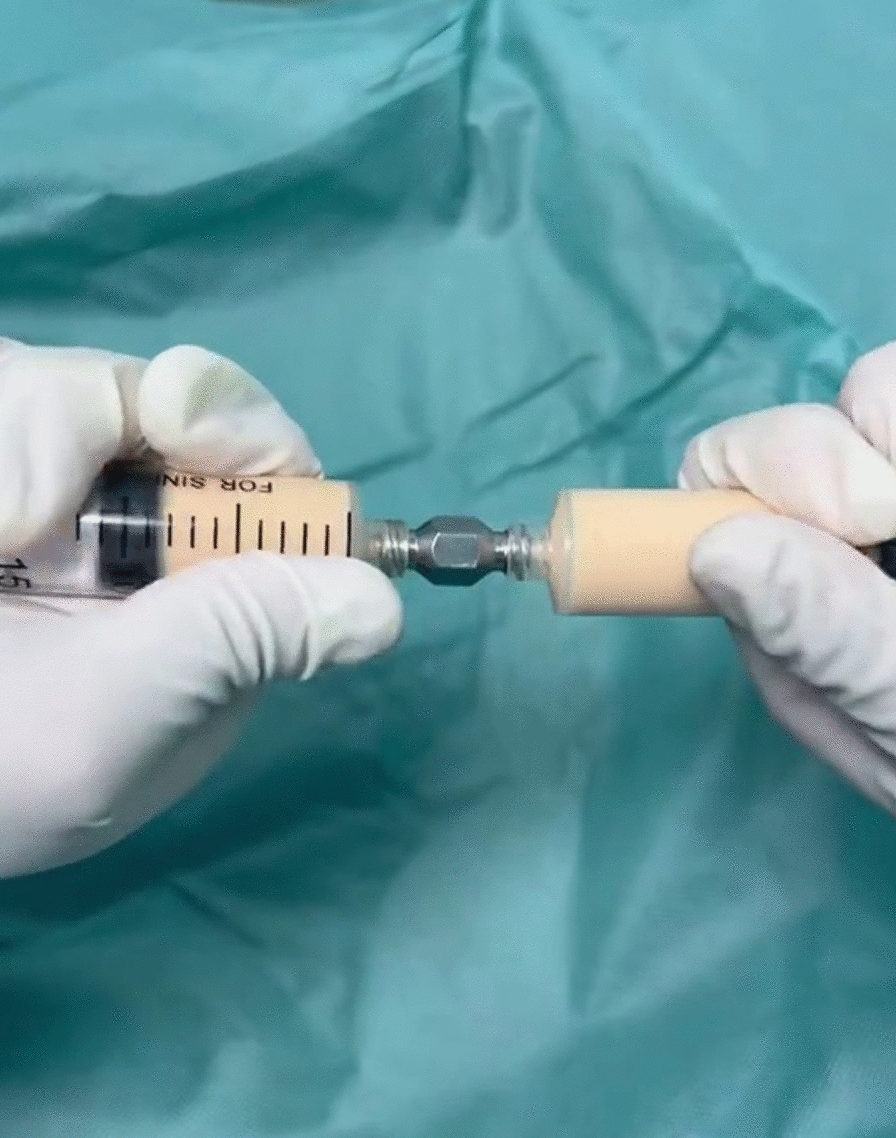
Mechanical emulsification process

**Fig. 3:**
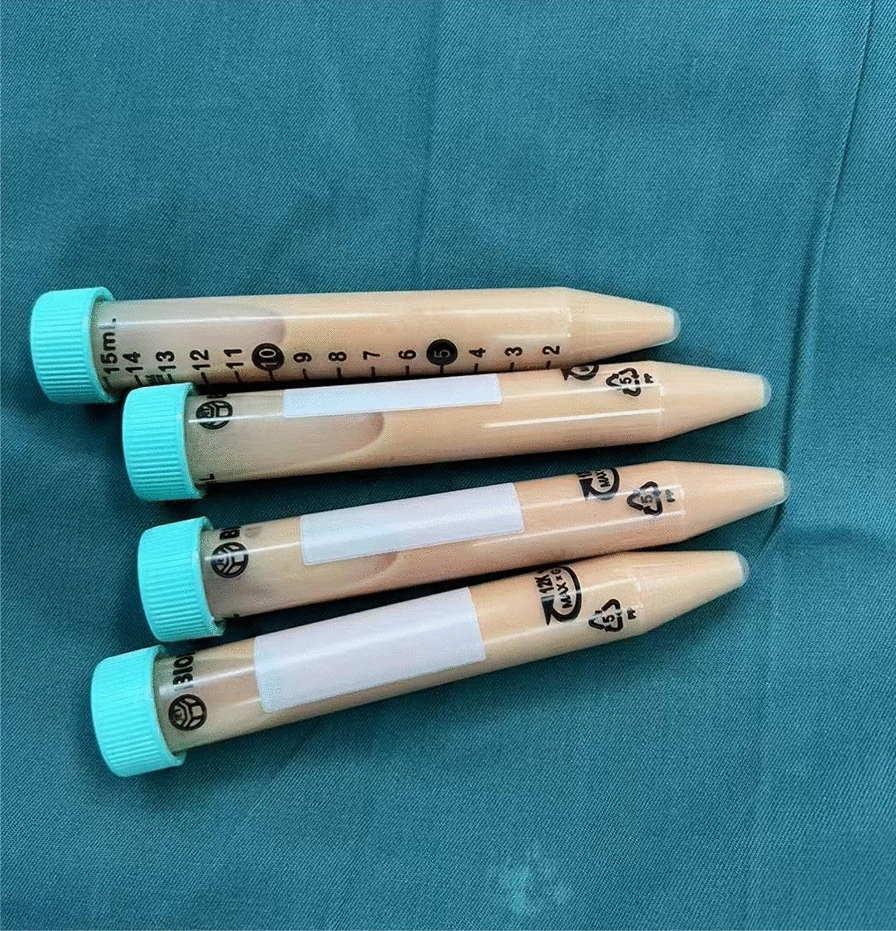
Adipose tissue after emulsification

**Fig. 4:**
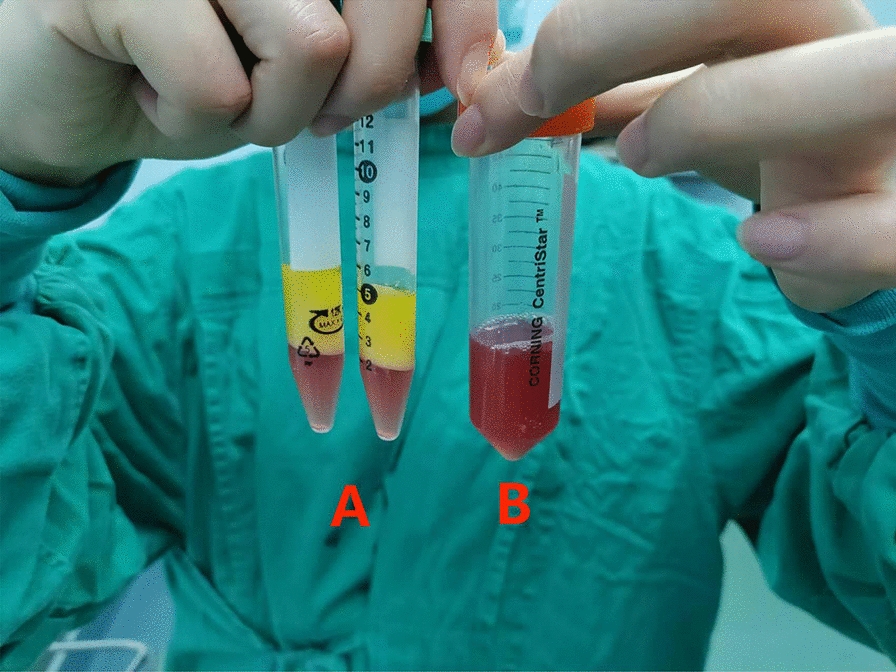
The letter (**A**) in the image indicates the stratification of emulsified adipose tissue after centrifugation, with the purified adipose liquid—a light red in color in the middle layer. The letter (**B**) in the image shows the cell-free fat extract obtained after further filtration of the purified fat solution

**Fig. 5:**
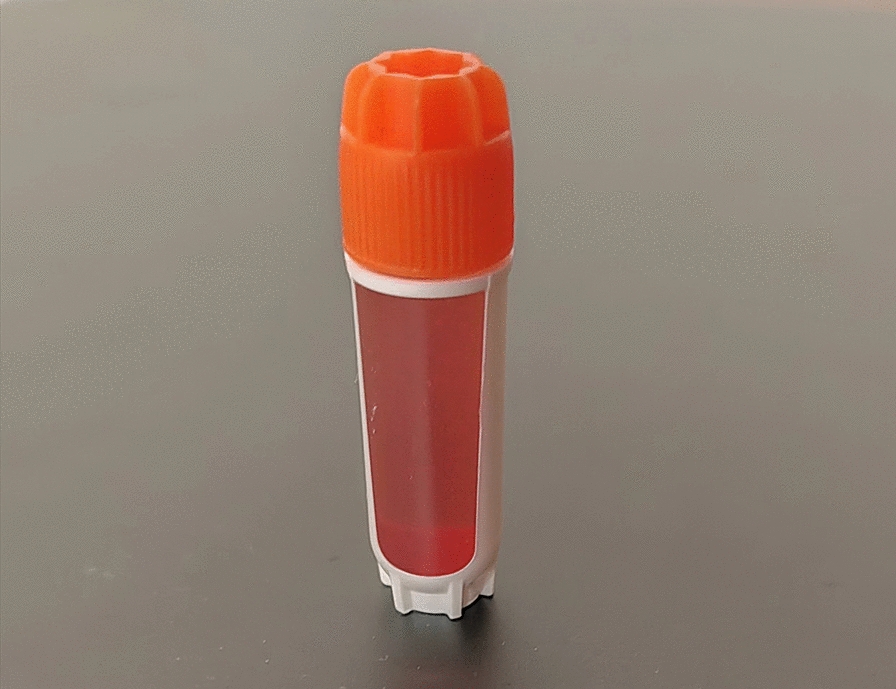
Cell-free fat extracts loaded into sterile freezer tubes (2 ml)

Similar to the preparation method of CEFFE proposed by Yu et al. [26], the process begins with disinfection, draping and local anaesthesia in the mid-abdomen of the subjects in the operating theatre by a surgeon from the Department of Plastic Surgery at our hospital. Subsequently, about 100 ml (unilateral knee) or 200 ml (bilateral knees) of adipose fluid was obtained by liposuction. The fluid was then put into saline for rinsing to remove the blood tissue. The washed adipose tissue was transferred into two 20 ml syringes connected by a Luer-Lok connector. Emulsification was then performed by pushing and pulling the two syringes back and forth 60 times (Fig. 2). The emulsified adipose tissue (Fig. 3) was centrifuged at 3000 r/min for 5 min in a centrifuge (Shanghai Lu Xiangyi Centrifuge Instrument Co., Ltd. TDZ5-WS), resulting in a three-layered liquid: an upper yellow oil layer, a middle layer of light-red purified fat liquid, and a lower layer of a small amount of clear white flocculent liquid (Fig. 4A). The middle layer was aspirated slowly using a syringe with a side-hole needle, and the cell-free fat extract was obtained by injecting the collected purified fat liquid into a 0.22 um filter for further filtration (Fig. 4B). The extract was then injected into 5 or 10 sterile 2 ml freezing tubes (Fig. 5), and stored at − 25 °C in a freezing environment. The entire process was performed under aseptic conditions.

### Intervention

**Fig.6:**
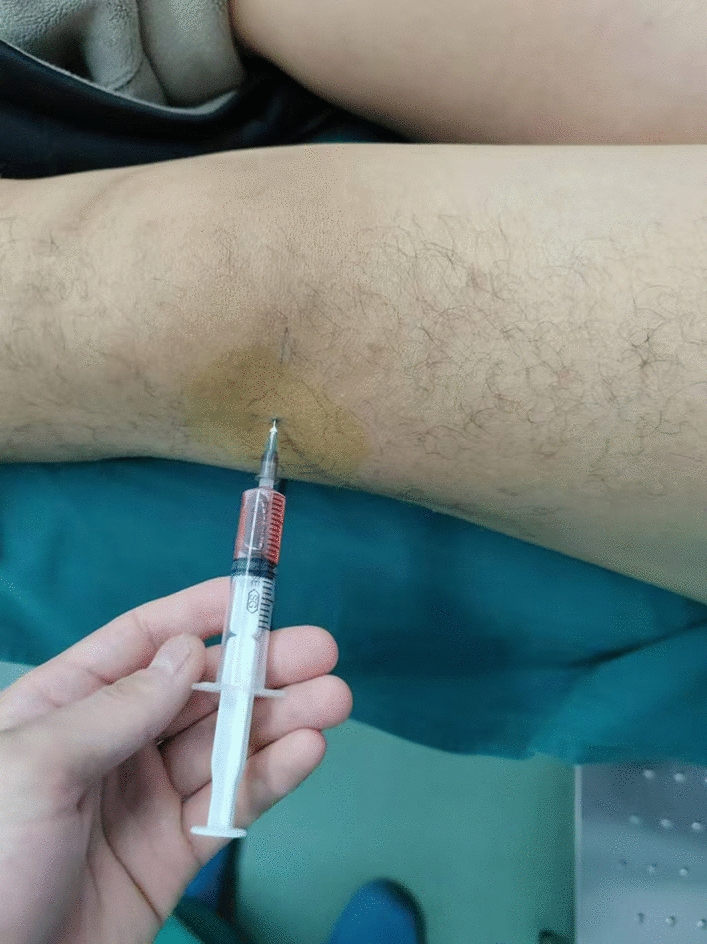
Intra-articular cavity injections of cell-free fat extracts

Intra-articular knee injections in both treatment groups were performed by experienced arthrologists who failed to involved in the subsequent follow-up evaluation. All subjects put their knees in an extended position, and after skin disinfection, an anterolateral approach to the knee was applied for arthrocentesis. In the experimental group, one frozen tube of CEFFE reagent was taken out of the refrigerator and thawed weekly; 2 ml of the reagent was then withdrawn from the syringe and injected intra-articularly for a total of 5 weeks (Fig. 6). In the control group, HA (Hyprojoint 1 ml/10 mg) was injected intra-articularly once a week with 2 ml of the reagent for 5 weeks. After the injection, the skin was re-sterilized and covered with an infusion patch. The patients were instructed to perform knee flexion and extension exercises 20 times to ensure the injection fluid evenly distributed within the joint cavity. Ice packs were applied to the puncture site for 2–3 h to relieve pain and temporary oral NSAIDs were prescribed as needed for intra-articular pain. During the treatment follow-up period, patients were informed not to take medications designed to nourish cartilage, improve joint function, or alleviate pain.

### Outcome measures

The following 3 methods were applied to assess the clinical effects of this trial by a specialized follow-up evaluator:

Clinical Rating Scales: subjects were assessed using three rating scale questionnaires at 5 time points: 1 day before treatment, 3 weeks after treatment (following the final injection), 6 weeks after treatment, 3 months after treatment, and 6 months after treatment. These rating scales included the VAS (visual analogue scale), the WOMAC (Western Ontario and McMaster Universities Osteoarthritis Index), and the Lysholm Knee Scale. The VAS [35], a pain analogue scale (0–10, with 0 indicating no pain and 10 indicating severe pain), assessed the degree of pain, with a lower score indicating less pain. The WOMAC scale[36], evaluated pain, stiffness, and functional limitations to assess the degree of joint pain and joint function, with a lower score indicating less pain and better joint function. The Lysholm Knee Scale [36] (0–100 points), included 7 observation items, such as limping, support, locking, joint instability, joint swelling, climbing stairs, squatting, and pain, with a higher score indicating better joint function.

Clinical efficacy: Referring to the clinical efficacy evaluation method of Wang Feng et al. [37], the clinical efficacy of the two groups was compared at 6 months post-treatment. Significant effect: the symptoms of knee joint pain, swelling and stiffness disappeared, with normal joint mobility. Effective: there was mild improvement in the symptoms of knee joint, with slight limitation in joint mobility. Ineffective: no improvement in the symptoms of knee joint or limitation of movement.

Bone marrow edema grading: Coronal, sagittal, and axial magnetic resonance images of the knee joint (layer thickness 4 mm, layer spacing 1 mm) were obtained by performing a 1.5 T knee MRI scan (Siemens MAGNETOM Skyra 1.5 T magnetic resonance imager) on the affected knee of the subjects, at both 1-day pre-treatment and 6 months post-treatment. According to the BME grading method for the knee joint by Felson et al. [38], joint bone marrow edema was assessed by observing the high-signal area on the adjacent subchondral bone in the femur and tibia on the coronal T2-weighted image. The femoral and tibial articular surfaces on the coronal T2-weighted image were then divided into six quadrants: medial, central and lateral (for both the femur and tibia). Each quadrant was graded based on the number of BME (high signal) regions on the image layer in the coronal T2-weighted image, which was categorized into four grades, grade 0 (no BME), grade 1 (BME on one or two consecutive image layers), grade 2 (BME on three consecutive image layers), and grade 3 (BME on four or more consecutive image layers) [38]. To simplify the classification of bone marrow edema, the above six quadrants were combined into three quadrants of the medial, central and lateral femoral-tibial joint, with a range of 0–6 grades. In other words, the severity of bone marrow edema is inversely proportional to the grade. The MRI grading of bone marrow edema was assessed by an experienced radiologist who was blinded to the study.

### Statistical analysis

Sample size calculation: the sample size was calculated using PASS software (version 21.0.3, developed by NCSS Corporation, USA). The expected difference between the means of the two groups was 10, with a standard deviation (SD) of 9. Assuming an alpha level (α) of 0.05, certainty of 0.90, and equal sample sizes in both groups (N1 = N2), the required sample size for each group was calculated to be 20 knees. Considering a potential dropout rate of 15%, a minimum of 23 knees were deemed necessary for each group, resulting in a total of at least 46 knees needed to be validly enrolled in the study.

Results analysis: The baseline characteristics of the enrolled patients and their knee joints, and the observed indicator data at each follow-up time point were tested for normality. Normally distributed continuous data are presented as the mean ± standard deviation, non-normally distributed data are presented as the median (upper quartile, lower quartile), and categorical data are presented as frequencies (percentages). For normally distributed continuous data, the *t*-test was used; for comparisons between two groups of non-normally distributed continuous data, the Mann–Whitney *U* test was employed, and for comparisons within groups, the Wilcoxon signed-rank test was utilized. The Pearson chi-square test or Mann–Whitney *U* test was applied to categorical data as appropriate. To assess the differences in non-normally distributed clinical scores at different follow-up time points, generalized estimating equations (GEE) analysis was first conducted to analyze the main effects and interaction effects of group and time. Subsequently, the nonparametric Mann–Whitney *U* test was used for comparisons between the two groups (group simple effect analysis). Friedman’s test was employed for overall comparisons of the time points within each group, and Wilcoxon signed-rank test was used for comparisons of the time points between the two groups (time simple effect analysis). A *P*-value < 0.05 was considered statistically significant in this study, and all statistical analyses were conducted as two-tailed tests. The data from this study were analyzed using SPSS statistical software, version 22.0 (IBM, Armonk, New York, USA).

## Results

In this trial with a follow-up period of 6 months, a total of 46 patients with mild-to-moderate knee osteoarthritis received intra-articular injections of medication, including 26 patients in the CEFFE group and 26 patients in the HA group. During the treatment period, 1 patient in the CEFFE group and 3 patients in the HA group developed joint swelling and pain after the injection, all of which were relieved within 2 days after local ice treatment. No other adverse phenomena and complications occurred in the remaining patients.

There were no statistically significant differences between the two groups in terms of gender, age, BMI, duration of pain, K-L grading of osteoarthritis in the affected limbs and knees, and pre-treatment VAS scores, WOMAC scores, Lysholm scores, bone marrow edema grading (*P* > 0.05) (Tables 1, 4).

**Table 1:** Comparison of baseline characteristics between the two groups

	CEFFE group (n = 26)	HA group (n = 26)	*P*
Number	23	23	–
Age (years)	53.60 ± 7.59	57.00 ± 8.88	0.150^a^
Gender (M/ F)	7/16	8/15	0.753^b^
BMI	23.90 ± 2.21	23.40 ± 2.99	0.454^a^
Duration of pain (months)	14.50 (12.00, 18.00)	12.00 (8.75, 20.50)	0.278^c^
Affected knee (left/right)	14/12	12/14	0.579^b^
K-L classification (II/III)	12/14	11/15	0.780^b^
WOMAC score	67.27 ± 4.46	65.38 ± 4.28	0.126^a^
VAS score	6.00 (5.00, 6.00)	6.00 (5.00, 6.00)	0.729^c^
Lysholm score	65.12 ± 6.18	63.27 ± 4.20	0.214^a^

CEFFE, cell-free fat extract; HA, hyaluronic acid; BMI, body mass index; K-L grading, Kellgren-Lawrence grading; WOMAC, Western Ontario and McMaster Universities Osteoarthritis Index; VAS, visual analogue scale

^a^comparison between groups was performed using *t*-test; ^b^comparison between groups was performed using  x^2^ test; ^c^comparison between groups was performed using z test

### Comparison of clinical scores

According to the generalized estimating equation (GEE) analysis (Tables 2), there were statistically significant differences in clinical scores (VAS, WOMAC, and Lysholm) between the two group at both the pre-treatment and post-treatment follow-up time points (*P* < 0.05). Comparing the changes in clinical scores of the time points within each group, significant differences were observed in both groups (*P* < 0.05). Among these founding, the group analysis of the main effects revealed that the differences in clinical scores between the two groups were statistically significant for all three clinical outcome measures (*P* < 0.05). Furthermore, the time analysis of main effects indicated that the clinical scores in both groups exhibited a trend of change over time (*P* < 0.05). Lastly, the interaction analysis of main effects between the three clinical score groups and time also showed statistically significant results (*P* < 0.05), suggesting that the two groups had different improvement trends in the clinical scores, with the CEFFE group demonstrating a more significant improvement compared to the HA group.

**Table 2:** Comparison of three clinical scores between and within two groups at different follow-up time points

	CEFFE group (n = 26)	HA group (n = 26)	*P* (intergroup)
*VAS*			
3W	4.00 (3.00,5.00)^*^	3.50 (3.00,5.00)^*^	0.433
6W	2.50 (2.00,3.00)^*a^	3.00 (3.00,4.00)^*^	0.040
3 M	2.00(1.00,3.00)^*ab^	3.00 (3.00,5.00)^*^	0.001
6 M	1.00 (1.00,2.25)^*ab^	3.00 (2.75,5.25)^*bc^	0.000
*P* (within-group)	< 0.001	< 0.001	
Group effect (Wald χ2, *P*)	6.376,0.012		
Time effect (Wald χ2,*P*)	404.007, < 0.001		
Interaction effect (Wald χ2,*P*)	27.533, < 0.001		
*WOMAC*			
3W	49.00 (39.50,55.00)^*^	46.50 (36.00, 55.25)^*^	0.790
6W	38.50 (32.75, 43.25)^*a^	45.50 (38.00,55.00)^*^	0.036
3 M	23.50 (22.00,48.00)^*ab^	44.00 (36.50,58.75)^*^	0.001
6 M	21.00 (17.75,46.50)^*ab^	44.50 (35.25,58.00)^*^	0.001
*P* (within-group)	< 0.001	< 0.001	
Group effect (F, *P*)	5.145,0.023		
Time effect (F,*P*)	213.962, < 0.001		
Interaction effect (F,*P*)	22.630, < 0.001		
*LYSHOLM*			
3W	80.00 (72.00, 85.00)^*^	78.00 (74.75, 82.00)*	0.137
6W	80.00 (72.00, 85.00)^*a^	78.50(72.75,80.00)^*^	0.001
3 M	90.00(78.00,91.00)^*ab^	79.00(64.75,82.00)^*^	< 0.001
6 M	90.00(80.25,92.00)^*ab^	79.00(62.50,82.75)^*^	< 0.001
*P* (within-group)	< 0.001	< 0.001	
Group effect (F, *P*)	13.915, < 0.001		
Time effect (F,*P*)	172.084, < 0.001		
Interaction effect (F,*P*)	16.418,0.003		

Data are provided as median (upper quartile, lower quartile); *P* (intergroup) of Mann–Whitney test; *P* (within-group) of the Friedman test

CEFFE, cell-free fat extract; HA, hyaluronic acid; VAS, visual analogue scale; WOMAC, Western Ontario and McMaster Universities Osteoarthritis Index; 3W, 3 weeks post-treatment; 6W, 6 weeks post-treatment; 3 M, 3 months post-treatment; 6 M, 6 months post-treatment

*^abc^comparisons of time points within the group using the Wilcoxon signed-rank test; *P < 0.05 compared to baseline; ^a^*P* < 0.05 compared to 3 weeks post-treatment; ^b^*P* < 0.05 compared to 6 weeks post-treatment; ^c^*P* < 0.05 compared to 3 months post-treatment

#### Comparison of clinical scores between groups

**Fig. 7:**
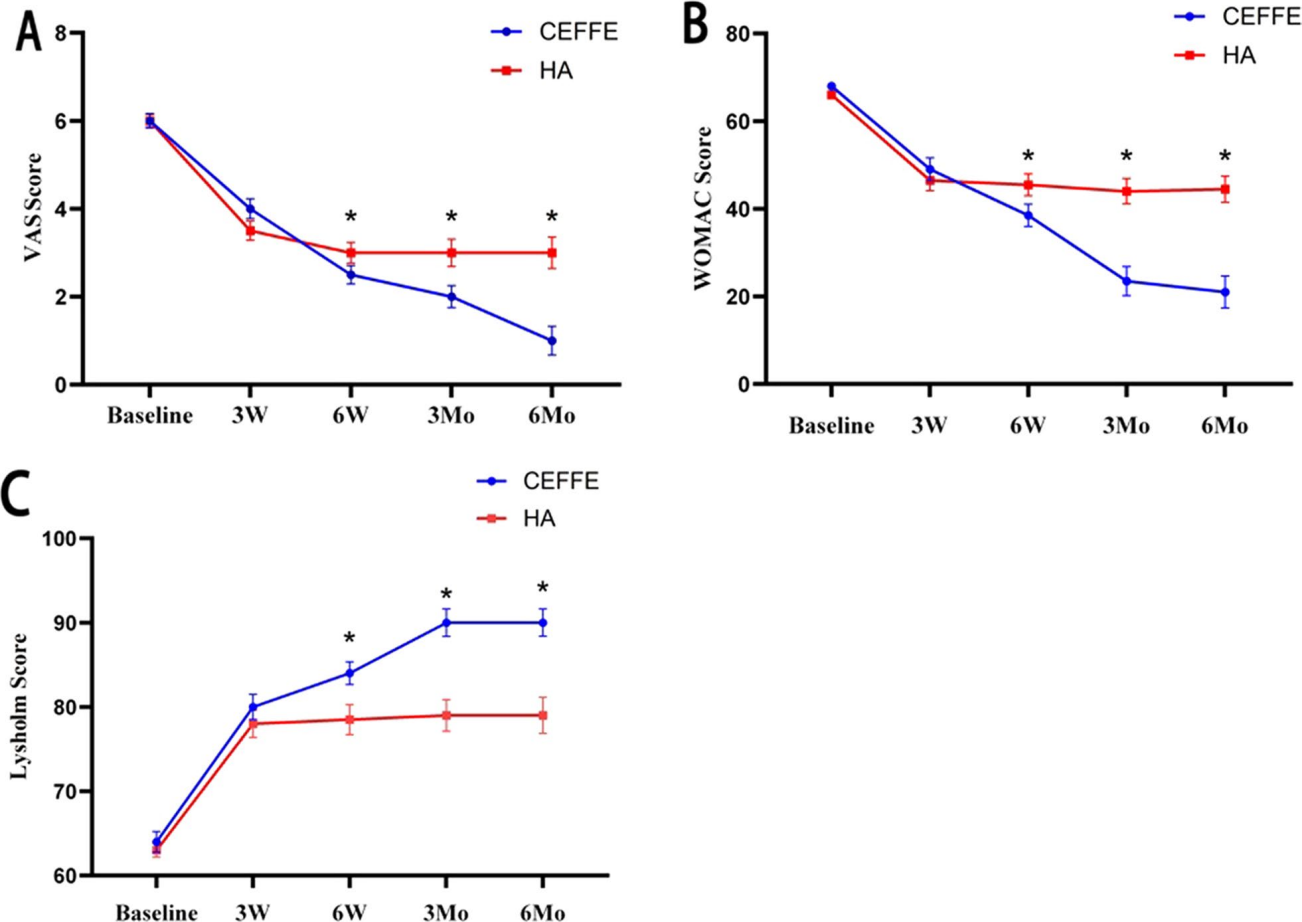
Comparison of VAS scores (**A**), WOMAC scores (**B**) and Lysholm scores (**C**) between the cell-free fat extract (CEFFE) group and the hyaluronic acid (HA) group at each follow-up time point. Data points are expressed as (median, standard error); ^*^represents *P* < 0.05 for two group comparisons; 3W, 3 weeks post-treatment; 6W, 6 weeks post-treatment; 3Mo, 3 months post-treatment; 6Mo, 6 months post-treatment

When comparing the VAS score, WOMAC score, and Lysholm score of patients in the two groups, the differences between pre-treatment and 3 weeks post-treatment were not statistically significant (*P* > 0.05). At 6 weeks, 3 months, and 6 months post-treatment, the VAS score and WOMAC score in the CEFFE group were lower than those in the HA group, while the Lysholm score in the CEFFE group was higher. All the differences were statistically significant (*P* < 0.05) (Table 2, Fig. 7).

#### Comparison of clinical scores within groups

##### CEFFE group

The results of pairwise comparisons at each follow-up time point within the CEFFE group were as follows. Compared to pre-treatment, VAS scores and WOMAC scores were lower and Lysholm scores were higher at each post-treatment time point (3 weeks, 6 weeks, 3 months, and 6 months) (*P* < 0.05). Compared to 3 weeks post-treatment, VAS scores and WOMAC scores at 6 weeks, 3 months, and 6 months post-treatment were lower, and Lysholm scores were higher, with all the differences being statistically significant (*P* < 0.05). Compared to 6 weeks post-treatment, VAS scores and WOMAC scores at 3 months and 6 months post-treatment were lower, and Lysholm scores were higher, with all the differences being statistically significant (*P* < 0.05). Compared to 3 months post-treatment, three clinical scores at 6 months post-treatment were not significantly higher or lower, with the differences not being statistically significant (*P* > 0.05) (Table 2, Fig. 7).

##### HA group

The results of pairwise comparisons at each follow-up time point within the HA group were as follows. Compared to pre-treatment, VAS scores and WOMAC scores were lower and Lysholm scores were higher at each post-treatment time point (3 weeks, 6 weeks, 3 months, and 6 months) (*P* < 0.05); In contrast to the CEFFE group, WOMAC scores and Lysholm scores in HA group did not show significant increases or decreases from 3 weeks to 6 months post-treatment. There were no statistically significant differences of the pairwise comparisons between the two groups at the four post-treatment time points (*P* > 0.05). Compared to 6 weeks post-treatment, VAS scores were higher at 3 weeks, 3 months, and 6 months post-treatment (*P* < 0.05). There were no statistically significant differences of the pairwise comparisons between the two groups at 3 weeks, 3 months, and 6 months post-treatment (*P* > 0.05) (Table 2, Fig. 7).

### Comparison of clinical efficacy

At 6 months post-treatment, the statistical analysis showed that the CEFFE group exhibited significantly better clinical efficacy compared to the HA group, with the difference being statistically significant (*P* < 0.05) (Table 3).

**Table 3:** Comparison of the clinical efficacy between the two groups

Groups	Significant effect	Effective	Ineffective	P*
CEFFE (n = 26)	17 (65.40)	5 (19.20)	4 (15.40)	0.008
HA (n = 26)	6 (23.10)	13 (50.00)	7 (26.90)	

Data are expressed as n (%), and the Mann–Whitney test was used for comparison between the two groups

CEFFE, cell-free fat extract; HA, hyaluronic acid

*comparison between groups was performed using Mann–Whitney test

### Bone marrow edema grading

**Fig. 8:**
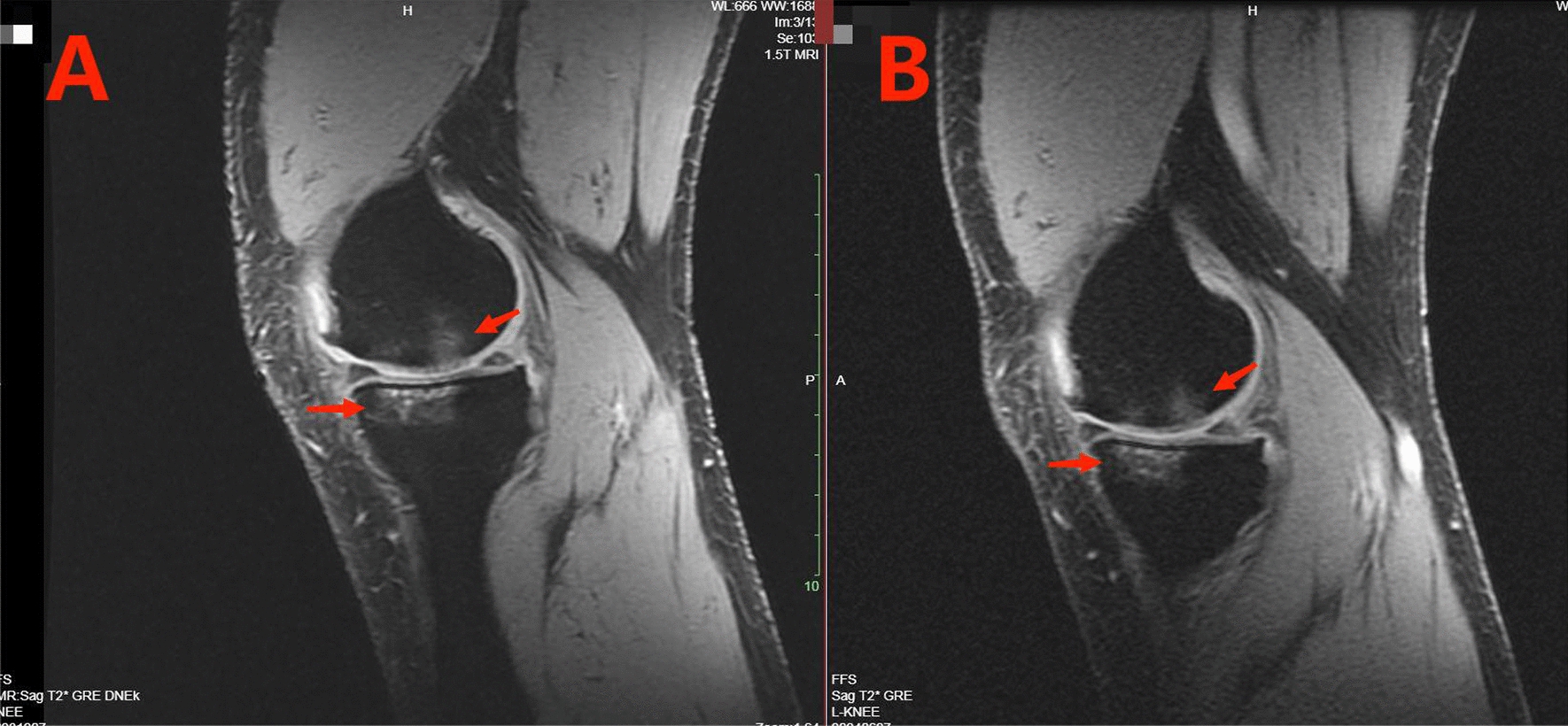
Pre-treatment (**A**) and 6 months post-treatment (**B**) MRI sagittal T2-weighted images of a 53-year-old male patient with osteoarthritis of the left knee. Subchondral bone marrow edema (indicated by arrows in the MRI image) was reduced at 6 months post-treatment compared to pre-treatment

Both groups underwent knee MRI scans at pre-treatment and 6 months post-treatment. The results showed that, compared with the pre-treatment, subchondral BME had been absorbed in both groups at 6 months post-treatment (Fig. 8). Specifically, the CEFFE group exhibited a reduction in bone marrow edema in the medial, central and lateral femoral-tibial joints, whereas the HA group showed a decrease only in the lateral and central femoral-tibial joints. The difference between the two groups was statistically significant (*P* < 0.05). When comparing the bone marrow edema grading between the two groups at 6 months post-treatment, it was found that the CEFFE group exhibited more significant reductions of the bone marrow edema in all three regions (medial, central and lateral femoral-tibial joints) compared to the HA group. The difference between the two groups was statistically significant (*P* < 0.05) (Table 4).

**Table 4:** Bone marrow edema grading at baseline and 6 months post-treatment in the two groups

	CEFFE group (n = 26)	HA group (n = 26)	P^*^
medial joint (Pre)	3.00 (2.00,4.00)	3.00 (3.00,4.00)	0.426
medial joint (6 m Post)	2.00(1.00,3.00)	3.00 (2.00,4.00)	0.001
*P*^a^	0.001	0.285	
Intermediate joint (Pre)	1.00(0.00,3.00)	2.00(1.00,3.00)	0.165
Intermediate joint (6 m Post)	1.00(0.00,1.00)	1.00(1.00,2.00)	0.006
*P*^a^	0.019	0.047	
Lateral joint (Pre)	2.00(1.00,3.00)	2.00(1.00,3.00)	0.902
Lateral joint (6 m Post)	1.00(0.00,1.00)	1.00(1.00,2.00)	0.018
*P*^a^	< 0.001	0.018	

Data are expressed as median (upper quartile, lower quartile); CEFFE, cell-free fat extract; HA, hyaluronic acid; Pre, pre-treatment; 6 m Post, 6 months post-treatment

*comparison between two groups; ^a^ comparison of bone marrow edema grading at baseline and 6 months post-treatment

## Discussion

We compared the clinical efficacy of intra-articular knee injections of CEFFE versus HA in patients with early to mid-stage knee osteoarthritis, as well as their effects on reducing bone marrow edema with a prospective, randomized controlled trial. This study demonstrated significant efficacy for both the CEFFE and HA groups in the treatment of early to knee mid-stage osteoarthritis. Both groups exhibited clinically significant improvements in joint function, pain, bone marrow edema, and quality-of-life enhancement during a follow-up period of 6 months. To assess the short-term clinical efficacy of the two groups, we conducted a follow-up at 3 weeks post-treatment and found no significant difference in pain relief and joint function improvement between the CEFFE group and the HA group in the immediate term. However, as the follow-up period extended beyond 6 weeks post-treatment, the CEFFE group exhibited a more significant improvement and superior clinical efficacy. Based on the line graphs and the results of pairwise comparisons at each follow-up time point, we observed that, compared with baseline, the clinical scores of the CEFFE group continued to improve over the three months following treatment, stabilizing thereafter until 6 months post-treatment with no further improvement. Similarly, the WOMAC and Lysholm scores of the HA group exhibited the most significant improvement within the first 3 weeks post-treatment, stabilizing subsequently until 6 months post-treatment without any additional improvement. Regarding the VAS score, it showed the most significant improvement at 6 weeks post-treatment, and then gradually increased over time, however, it remained significantly lower than the pre-treatment level even at 6 months post-treatment. This trajectory of pain relief is similar to the results of the meta-analysis by Bannuru RR et al., which concluded that the effect of intra-articular HA injections for the treatment of pain symptoms in knee osteoarthritis (KOA) peaks at 8 weeks post-treatment, then tapers off, yet still offers some relief at 24 weeks post-treatment [39]. Based on this study and previous related research [40, 41], the duration of HA in treating osteoarthritis symptoms is at least 6 months, and we can infer that the duration of CEFFE in treating osteoarthritis symptoms exceeds 6 months and is longer than that of HA. This suggests that intra-articular injection of CEFFE offers unique advantages for the long-term pain relief in patients with KOA. However, longer follow-up periods are necessary to validate these findings. Consequently, the distinct clinical efficacy trajectories, duration and intergroup differences in the results of the two groups in this study require further exploration of underlying causes.

With the in-depth study of osteoarthritis, it has been found that articular cartilage plays a crucial role in its development [42]. The degeneration and regeneration of cartilage are closely related to the relief of clinical symptoms. Currently, for the conservative treatment of early to mid-stage knee osteoarthritis, intra-articular injection of platelet-rich plasma (PRP) or hyaluronic acid (HA) has proven to have reliable therapeutic effects and application value [43]. HA primarily functions as a lubricant, shock absorber in the knee joint, facilitating the regeneration of endogenous hyaluronic acid, thereby effectively slowing down the decomposition of cartilage matrix, the wear and tear of articular cartilage, and metabolic abnormalities in the subchondral bone, including edema, hemorrhage and sclerosis [44, 45]. Numerous studies have demonstrated the palliative effects of intra-articular injection of hyaluronic acid in the treatment of early to mid-stage knee osteoarthritis, which can reduce pain, improve knee function, and reduce the edema of the articular subchondral bone, and the promote joint effusion [46–48]. However, HA’s role as a lubricant gradually diminishes over time [46], requiring multiple intra-articular injections, which also increases the risk of infection following total knee arthroplasty [49]. Consequently, the development of a new reagent with a prolonged duration of action has emerged as a therapeutic trend.

Cell-free fat extract (CEFFE) is a non-immuno-rejecting, sterile, ethically compliant, multifunctional biologic obtained by mechanical emulsification of autologous adipose tissue isolation. It contains a variety of factors including brain-derived neurotrophic factor (BDNF), transforming growth factor-β (TGF-β), hepatocyte growth factor (HGF), basic fibroblast growth factor (B-FGF), vascular endothelial growth factor (VEGF), platelet-derived growth factor (PDGF), epidermal growth factor (EGF), and neurotrophic factor-3 (NT-3). These growth factors play crucial roles in promoting tissue regeneration and repair, anti-apoptosis, antioxidant protection, and proliferation [26, 30, 32].

Meanwhile, it has been found that these growth factors within CEFFE can increase the proportion of anti-inflammatory M2 macrophages in joint tissues and promote the expression of ARG, IL-10 anti-inflammatory factors, and type II collagen. Additionally, it can decrease the proportion of pro-inflammatory M1 macrophages and inhibit the expression of inflammatory factors, such as IL-1β, IL-6, iNOS, NO, and TNF-α, as well as MMPs. Elevated levels of certain inflammatory factors can exacerbate the clinical symptoms of joint pain and stiffness, and accelerate the destruction of cartilage [50, 51]. Matrix metalloproteinases (MMPs) are implicated in the degradation of cartilage matrix and inhibition of type II collagen synthesis in osteoarthritis [52]. Furthermore, CEFFE reduces the expression of inflammatory factors by scavenging and lowering the levels of reactive oxygen species (ROS) [53], ultimately promoting injured cartilage synthesis and mitigating cartilage degradation. Therefore, by regulating the interplay between anti-inflammatory and pro-inflammatory factors, CEFFE improves the inflammatory microenvironment in the joints, promotes cartilage synthesis, relieves clinical symptoms, and slows down the progression of early to mid-stage osteoarthritis.

BME is a manifestation of cartilage damage in the subchondral bone during the early stages of osteoarthritis, resulting from the effect of mechanical stimulation and inflammatory mediators. It serves as a marker to predict the onset and progression of osteoarthritis, and appears as a high signal lesion in bone marrow on T2-weighted MRI scans [33]. Studies have shown that the occurrence of painful symptoms in patients with knee osteoarthritis (KOA) is closely related to BME, with the severity of knee pain being directly proportional to the area size of the BME [54]. To assess the severity of BME in the knee, Felson et al. graded the MRI images based on the area size of BME and the number of image layers across the six articular surface quadrants [38]. However, the present study has simplified the data collection and statistical process by consolidating these into three articular surface quadrants. Studies have shown that BME is associated with inflammatory factors released within the subchondral bone microenvironment [55], and CEFFE contains a variety of growth factors that can exert an anti-inflammatory effect to control the progression of intra-articular BME and alleviate pain symptoms. Therefore, comparing the MRI results of CEFFE and HA at pre-treatment and 6 months post-treatment showed that the subchondral BME of patients in both the CEFFE and HA groups decreased to varying degrees compared with pre-treatment levels. The results of intergroup comparison showed that the reduction of BME in the CEFFE group was significantly better than that in the HA group. In contrast to the CEFFE group, after 6 months of treatment, the HA group with no significant improvement in medial femoral-tibial joints bone marrow edema. This may be related to the anatomical differences between the medial and lateral femoral condyles of the knee joint, where the larger structure of the medial condyle subjects the medial side of the knee joint to greater stress. Consequently, some studies have shown that in the long-term application of knee joint, the degree of chondral wear and tear on the medial joint surface is more severe than on other joint surfaces [56], leading to a more pronounced occurrence of bone marrow edema on the medial joint surface. However, the lubricating effect of HA may diminish with prolonged treatment [46], gradually weakening its effect on reducing joint wear and subchondral bone edema (bone marrow edema). Finally, we found that the reduction in BME at 6 months post-treatment was consistent with the results of the VAS score.

This study has several limitations. Firstly, our trial conducted with a relatively small sample size, which may compromise statistical significance and impact the generalization and reproducibility of the findings. Furthermore, the follow-up period was relatively short, hindering the exploration of long-term clinical effects. Consequently, future investigations ought to focus on enlarging the sample size and prolonging the follow-up duration. Secondly, the preparation process of CEFFE deviated slightly from that described by Yu et al. Due to economic and equipment limitations, this study omitted the secondary centrifugation and thawing steps. Additionally, variations in centrifugation speed, time, the number of mechanical emulsification cycles, and freezing temperatures existed, and concentration errors in the prepared reagents may influence their clinical efficacy. Thirdly, this study only statistically analyzed the WOMAC total score index and neglected the three sub-scores: WOMAC pain, WOMAC stiffness, and WOMAC function. This omission precluded a direct assessment of the therapeutic effect on joint pain, stiffness, and functional symptoms. Future studies should emphasize the comprehensiveness and relevance of the observational indicators. Fourthly, the KL grading system only encompassed grades II/III, excluding grade I early osteoarthritis. Moreover, some grade III patients were already in the advanced stage of KOA, leading to suboptimal treatment outcomes for both groups of reagents, which may undermine the credibility of the observational indicators results.

## Conclusion

This study demonstrated that intra-articular injection of CEFFE could modulate the inflammatory microenvironment in the joint, enhance the durability of tissue-specific cells (especially chondrocytes), and improve cellular metabolic processes. Consequently, it reduces further degeneration of the knee joint and prevents the continued progression of osteoarthritis. Additionally, both CEFFE and HA exhibited significant clinical improvement in patients with early to mid-stage knee osteoarthritis, with both treatments reducing subchondral bone marrow oedema. Notably, CEFFE was found to be more effective than HA in this regard. Furthermore, the duration of clinical improvement was greater than 6 months for both treatments, with CEFFE demonstrating a longer-lasting effect compared to HA.

## Data Availability

Data are available via the corresponding author under a reasonable request.

## Abbreviations

CEFFE: Cell-free fat extract
HA: Hyaluronic acid
KOA: Knee osteoarthritis
MRI: Magnetic resonance imaging
VAS: Visual analogue scale
WOMAC: Western Ontario and McMaster Universities Osteoarthritis Index
BME: Bone marrow edema
BMI: Body mass index
ELISA: Enzyme linked immunosorbent assay
NSAIDs: Nonsteroidal anti-inflammatory drugs
MIA: Sodium iodoacetate
MMP: Metalloproteinase
PRP: Platelet-rich plasma
NOS: Nitric oxide synthase
ALT: Alanine transaminase
AST: Aspartate aminotransferase
CR: Creatinine
BUN: Blood urea nitrogen
GFR: Glomerular filtration rate
PT: Prothrombin time
PTT: Partial thromboplastin time
HBsAg: Hepatitis B surface antigen
HIV Ag/Ab: Human immunodeficiency virus antigen/antibody
